# Stress and job satisfaction among nursing faculties in academic institutions in Nepal: A cross-sectional comparison between government and private institutions

**DOI:** 10.1371/journal.pone.0327657

**Published:** 2025-07-10

**Authors:** Jamuna Bhattarai, Muna Rana, Subhadra Khakurel, Usha Yadav, Heema Sunar

**Affiliations:** 1 Birgunj Nursing Campus, Institute of Medicine, Tribhuvan University, Birgunj, Nepal; 2 Maharajgunj Nursing Campus, Institute of Medicine, Tribhuvan University, Kathmandu, Nepal; 3 Tribhuvan University Teaching Hospital, Maharajgunj, Kathmandu, Nepal; 4 Department of Health Science, Ministry of Health and Population, Kathmandu, Nepal; COMSATS University Islamabad - Wah Campus, PAKISTAN

## Abstract

**Background:**

Stress and job dissatisfaction are significant factors leading to reduced efficiency and loss of human resources in all sectors, especially nursing education. This study aimed to compare the levels of stress, and job satisfaction among nursing faculty members in government and private colleges in Nepal.

**Methods:**

A cross-sectional study design was employed and conducted across nursing colleges in both the government and private sectors of Nepal. A total of 576 faculties were conveniently recruited from 13 government colleges and 26 private colleges for this study. The data were summarized using descriptive and inferential statistics, comparing the results between the faculties of the government and the private colleges.

**Results:**

Among the 576 respondents, 24.7% reported high levels of stress, while the majority (62.7%) were ambivalent about their job and less than one-third were satisfied. The majority of the respondents working in government colleges reported lower stress levels. Regarding job satisfaction, 8.9% of respondents were dissatisfied, and 30.7% were satisfied in government colleges, which was 2% higher than in private colleges. More than half of the respondents were ambivalent about their jobs in both private (63.7%) and government colleges (60.3%). The levels of stress, and job satisfaction did not significantly differ between government and private colleges.

**Conclusions:**

Stress levels are slightly higher in private colleges, while faculty members in government colleges report greater job satisfaction. Future research could be directed towards the identification of ways to reduce stress and promote satisfaction among nursing faculties, especially in private colleges.

## Introduction

Employee turnover is a significant concern for organizations, as it is detrimental to both the organization as well as the employees. The high turnover rate of nursing professionals is a global issue [1]. Nursing is a stressful occupation, and nurses experience high levels of burnout, which is strongly associated with work-related stress [2,3]. The mismatch between job expectations and actual working conditions contributes to higher levels of stress and poor job satisfaction. Research indicates that job stress among health personnel, including nurses, is alarming and adversely affects their health. Nursing personnel, who provide patient care as well as work in nursing academia, experience a double burden of stress [4]. A range of factors influence job satisfaction, and understanding these factors helps to enhance it. Satisfied employees are more likely to remain in their jobs, reducing the shortage of a diverse academic workforce [4]. Studies have shown that empowering nurses increases their job satisfaction, leading to improved patient care and safety, and also student satisfaction. Key research findings indicate that personnel, organizational, managerial, academic, professional, and economic variables are related to job satisfaction, while stress and burnout were associated with lower job satisfaction [5].

Effective higher education requires a satisfied workforce to achieve the highest academic goals for the organization [4]. Academia should foster an environment that promotes the psychological empowerment of faculties, enabling them to be self-directed, productive, confident, and find a meaningful connection to their work [2]. However, findings indicate that nurse educators had higher levels of burnout and required more support than staff nurses [6].

The future of the nursing workforce depends on qualified nursing faculty to educate nursing students [7]. According to the National Advisory Council on Nurse Education and Practice (NACNEP), the shortage of nursing faculty in the United States and other high-income countries limited their ability to produce more nurses. A key reason for this shortage is the inability of the colleges to accept new applications for student nurses due to faculty shortages [8]. Other factors contributing to this shortage include a lack of master’s and doctorate-prepared nurses, an ageing faculty workforce, difficulty recruiting younger faculty, low salaries, and decreased job satisfaction [9]. Specific factors affecting job satisfaction include a lack of knowledge related to the faculty role, challenges in sustaining and funding nurse faculty programs, role transition difficulties, job stress, and maintaining work-life balance [8,10,11].

Measuring the stress and level of job satisfaction of university academicians can be linked to various organizational outcomes, including employee misbehaviour, job performance, turnover, stress, absenteeism, supervision, salary, promotion, opportunities, completed tasks, communication, and coworker relationships [12]. Work-related stress from academic stressors is associated with poor job satisfaction. Research indicates a high level of job satisfaction was associated with low burnout levels [3]. Research in South Africa among nurses found a relationship between work-related stress, burn-out, job satisfaction, and general physical and mental health of nurses, which compromises the productivity, performance as well as quality of patient care [2]. Factors such as job-related stress, availability of psychological empowerment, tenure status, and mentoring quality significantly influence job satisfaction [13].

Historic strikes by nurses in the UK’s NHS highlight issues of low pay, poor staffing, and working conditions, reflecting struggles faced by nurses and nursing academicians in developed nations [14–21]. The situation in developing countries, particularly Nepal, is even more challenging [22–25].

Recent global studies have highlighted the significant impact of workplace stress and job satisfaction on nursing faculty, particularly in academic settings. For instance, a study by Alharbi et al. found that nursing faculty in higher education institutions often experience high levels of stress due to heavy workloads, administrative responsibilities, and lack of institutional support, which negatively affects their job satisfaction [26]. Similarly, recent evidence indicates that job satisfaction among nursing faculty in Nepal remains a significant challenge, with studies revealing concerning levels of dissatisfaction within academic institutions. A comprehensive cross-sectional study by Sapkota et al. found that only 36.8% of nursing faculty across multiple universities in Nepal reported satisfaction with their current positions [27]. This study identified two critical factors significantly associated with job satisfaction: involvement in departmental decision-making processes (OR = 4.83) and adequate access to reference materials (OR = 2.90) [27]. The findings align with global trends highlighting the importance of autonomy and resource availability in faculty satisfaction. Additionally, research has revealed a critical shortage of qualified public health faculty in Nepal, with many instructors teaching multiple subjects despite limited formal education in those areas [28]. This shortage inevitably contributes to increased workload and stress levels among existing faculty members.

Further investigation into the Nepalese context reveals notable differences in job satisfaction between government and private institutions. Recent research by Poudel examining healthcare professionals in eastern Nepal found that job satisfaction levels were significantly higher among those working in government institutions compared to their counterparts in private settings [29]. This disparity was primarily attributed to differences in salary structures and employment security between the two sectors. The study reported that 54.5% of nurses overall were satisfied with their positions, while 45.5% expressed dissatisfaction [29]. These findings are particularly relevant when examining the academic nursing environment, as they suggest institutional differences may significantly impact faculty well-being and performance. Addressing these disparities through enhanced teaching resources, opportunities for professional development, and greater involvement in institutional decision-making could substantially improve job satisfaction and reduce stress among nursing faculty across Nepal.

Research on stress among nursing faculty often uses Gmelch’s Faculty Stress Index (FSI) to measure job-related stress. A significant study in the United States involving 959 full-time nursing faculty used Gmelch’s FSI, Dreher’s mentoring scale, and Spreitzer’s psychological empowerment scale to assess job stress, mentoring quality, psychological empowerment, and job satisfaction. This study found that higher job stress was associated with lower job satisfaction, while effective mentoring and psychological empowerment positively influenced job satisfaction and reduced stress levels [10]. Similar studies in Israel have adapted the FSI to understand academic stress, identifying common stressors such as workload, institutional support, and mentoring quality [30]. These studies consistently highlight that strong mentoring and institutional support can mitigate stress and enhance job satisfaction among faculty members. Using Gmelch’s FSI, researchers can measure stress levels and identify specific stressors, facilitating targeted interventions to improve faculty well-being and retention. Comparing results across different countries helps understand global commonalities and unique challenges in faculty stress, informing more effective and culturally relevant strategies to support nursing faculty worldwide.

Research on job satisfaction among nursing faculty using Spector’s Job Satisfaction Survey (JSS) has been conducted in various countries, including the United States and Malaysia. These studies analysed key variables such as pay, promotion, fringe benefits, supervision, coworkers, nature of work, and communication. In Malaysia, significant differences in job satisfaction levels were found compared to Singapore and the United States, particularly in areas like pay and promotion, underscoring the impact of cultural and contextual factors [31]. In another study in the United States, how different leadership styles influenced job satisfaction among faculty members was analysed, which showed that transformational and transactional leadership styles were associated with higher job satisfaction, while passive/avoidant styles led to decreased satisfaction [32]. These findings illustrate the broad applicability and utility of Spector’s JSS in assessing job satisfaction across diverse cultural and organizational contexts, enabling researchers to identify critical factors influencing job satisfaction and informing targeted interventions to improve faculty well-being and retention.

In Nepal, 12 universities provide higher education, established through Acts approved by Parliament and functioning as public institutions namely, Tribhuvan University (1959), Nepal Sanskrit University (1986), Kathmandu University (1991), Purbanchal University (1994), Pokhara University (1997), Lumbini Bouddha University (2005), Far-western University (2010), Mid-western University (2010), Agriculture and Forestry University (2010), Rajarshi Janak University (2017) Madan Bhandari University of Science and Technology (2022) Among these, Tribhuvan University, Kathmandu University, Purbanchal University, and Pokhara University have provisions for nursing education from the bachelor level to Ph.D. [33]. Specialized institutions in Nepal, such as the National Academy of Medical Sciences (NAMS, 2002), BP Koirala Institute of Health Sciences (BPKIHS, 1993), Patan Academy of Health Sciences (PAHS, 2008), and Karnali Academy of Health Sciences (KAHS, 2011), play a crucial role in providing nursing education. These institutions are recognized for their contributions to medical and nursing graduate and postgraduate education, with a focus on community-based healthcare and research [34–37]. The Council for Technical Education and Vocational Training (1989) is a national autonomous apex body of Technical and Vocational Education and Training. Proficiency Certificate Level of Nursing and Auxiliary Health Midwifery Programme are incorporated under this [38]. The Medical Education Commission is the national regulatory body for Health Professional Education in Nepal [39].

The number of nurses produced in Nepal is increasing due to the growing number of nursing colleges and the popularity of nursing as a career. A total of 74,840 nurses are registered in the Nepal Nursing Council as of December 04, 2017 [40]. There are a total of 150 nursing colleges that are accredited by the Nepal Nursing Council with an estimated 2,761 faculty members that represent 3.68% of the total nursing workforce [40]. Only 15 of these colleges are government owned and the rest are private.

Nurses in low-income nations, particularly Nepal, are among the most overworked, underpaid, and underappreciated professionals. Nurses provide holistic care, including personal counselling, and 24 hours of patient recovery support, which includes promotive, preventive, and clinical care. Despite their crucial role in patient recovery, they are paid a small fraction of what doctors are paid and are treated with minimal respect by patients and hospital administration [41]. The government struggles to organize and distribute the growing pool of trained nurses all over the country, leading to an increasing migration of qualified and unemployed nurses to other countries [23–25], including nurses who are dissatisfied with their jobs [42]. Nurses in both clinical and academic settings suffer from burnout due to long working hours as well as job insecurity, coupled with low pay and lack of benefits, especially in private institutions.

Given the working facilities and organizational structure are different in government and private colleges in Nepal, we hypothesized that the stress levels and job satisfaction differed between the nursing faculty members in these two sectors. Hence this study aimed to compare the levels of stress and job satisfaction among faculty members in government and private institutes and to examine their association with organizational variables. However, much of the research in this area so far is focused on clinical settings, with little attention from concerned authorities. There are no baseline data available about this phenomenon among nursing personnel working as academic faculty in Nepal.

Interventions to reduce occupational stress are necessary to reduce burnout among nurses [43]. Effective strategies to support physical and mental health, such as employment support and stress management training, are crucial for enhancing nurses’ physical and psychological well-being [44]. The study’s findings can help stakeholders develop strategies to mitigate job dissatisfaction and stress among nursing faculties.

## Materials and methods

### Study design

This study employed a cross-sectional study design using a survey method of data collection from participants who responded to a questionnaire once during the research period.

### Study population and sampling

An estimated 2761 nursing faculties were working in different nursing colleges in Nepal, including an estimated 300 faculties working in government colleges.

Taking a census approach to cover all faculty members in the government, which is 300, a similar number of participants were expected to be recruited from private nursing colleges. So, all government colleges were included in the study, and a convenience sampling technique was used to select the private institutions. Similarly, the private institutions in the same administrative region as the government colleges and providing at least a diploma or a bachelor’s degree in nursing education, were conveniently selected. This way, the study aimed to recruit 600 participants (300 each from government and private colleges) for the research.

### Inclusion and exclusion criteria

Inclusion criteria for respondents in the study were namely, graduate nurses involved in academia, who had completed at least one year of teaching in the same institute on a full-time basis, and those who were willing to participate in the study. The exclusion criteria for respondents were those who were absent from work during the data collection period, and who were not willing to participate in the study.

### Research instrument

The research instrument was divided into three sections:

- Part I included sociodemographic and organization-related information. This section was constructed by the researchers through a literature search and discussion with the experts.
- Part II of the questionnaire was designed to collect information on stress. Stress was measured by Gmelch’s Faculty Stress Index, which was used with some adaptation after obtaining permission. There was a total of 36 statements, each question included 6-point Likert scale responses: not applicable, very slight pressure, slight pressure, moderate pressure, high pressure, excessive pressure [45].
- Part III of the questionnaire was designed to collect information regarding job satisfaction. This part consisted of a 36-item ‘job satisfaction survey’ by Spector and was used with permission. Each question here also included 6-point Likert scale responses (1: disagree very much, 2: disagree moderately, 3: disagree slightly, 4: agree slightly, 5: agree moderately, and 6: agree very much). These questions covered nine different domains and included four questions in each domain [46].

### Ethical approval

Ethical approval for the study was received from the Ethical Review Board, Institute of Medicine, Tribhuvan University, Nepal [Letter No: 442(6-11) E^2^ 075/76] on May 17, 2019. Written permission was taken from the colleges where the faculties were working. In each participating college, one faculty member was allocated to coordinate with researchers for data collection. The facilitators were provided with a remuneration of NPR 500.00 (~3.6 USD) to compensate for their time spent during the coordination of the data collection.

### Participant recruitment and data collection procedure

We collected the email addresses of all faculties from the coordinator at each college. In total, 600 faculty members in 41 participating colleges (15 government and 26 private colleges) were contacted with an invitation to participate in the research and informed consent pack through email. All participants provided a written information sheet and written informed consent was obtained before their enrolment into the study. Once the signed consent form was received, participants were asked to fill out the questionnaire through a Google form. The informed consent pack included a detailed information sheet that briefly described brief description of the study and research methods, sufficient information, and assurances about taking part to allow participants to understand the implications of participation and to reach a fully informed and freely given decision about whether or not to do so, without the exercise of any pressure or coercion. Reiterated the participant’s right to withdraw from the research project at any time without penalty.

**Fig 1:**
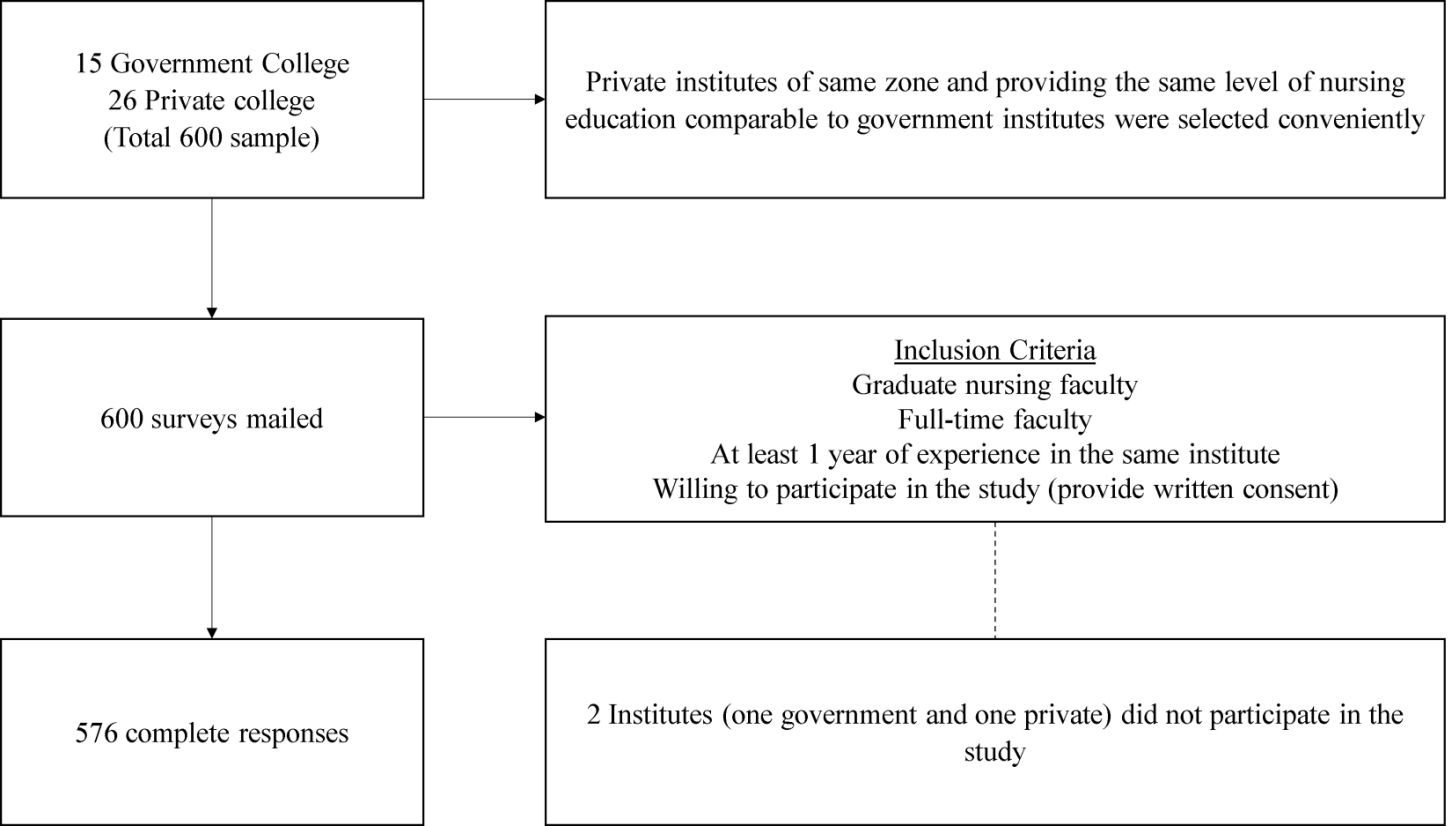
Participant recruitment and data collection process.

As two colleges did not respond, we only received a response from 39 colleges, which resulted in 576 responses. The anonymity of individuals and organizations participating in the research was ensured (Fig 1).

### Data analysis

The collected data were organized, coded, and exported to SPSS version 16 for analysis. We calculated the mean scores of stress and job satisfaction for faculties in government and private institutes followed by the determination of percent scores. We used a mid-value cut-off to classify stress levels as high or low after consultation with the stress scale author. For job satisfaction, we analysed the scores as per the scale developers’ guidance with the mean scores between 3 and 4 considered ambivalent. Summed scores for the 4-item subscales ranged from 4 to 24, with 4–12 indicating dissatisfaction, 16–24 indicating satisfaction, and 12–16 being ambivalent. For the 36-item total, scores ranged from 36 to 216, with 36–108 indicating dissatisfaction, 144–216 indicating satisfaction, and 108–144 being ambivalent [46].

Descriptive statistics (frequency, percentage, mean, and standard deviation) were used to summarize the data. The chi-square test was used to compare stress and job satisfaction levels. Association between selected socio-demographic variables with stress, and job satisfaction between faculties of two sectors were tested using the Chi-square test at 95% confidence interval and 5% level of significance.

## Results

The response rate was calculated at 96% (576 out of 600). Among 576 participants, 50.5% were of the age group 31–40 years. The mean age was 35.05 years, and the majority (98.3%) were female. More than one-third (38.0%) were from the Brahmin caste and around two-thirds (64.9%) of the respondents had completed a master’s degree in nursing. Regarding marital status, 86.8% were married.

Regarding the organizational factors of respondents, 68.9% were from private organizations and the remaining (31.1%) were from government organizations. Around half had the experience of 1–5 years in teaching. More than half (53.5%) belonged to the Assistant Professor/Lecturer academic job level and 61.8% were involved in the teaching of Bachelor of Nursing. Income ranged from NRs.16,000–200,000 per month and the mean income was NRs. 44,454 per month. Most of the respondents (76.9%) worked 7 hours a day. Around half (53.1%) said that the organization had no provision for higher education, however, the majority said that there was an opportunity for training and leave. Among all, 65.8% were permanently tenured and 34.2% were in temporary status.

Table 1 presents the reason and level of stress felt by respondents. Among all, 36% of the respondents said that participating in the work of departmental or university committees is not applicable in their workplace, whereas 1.7% felt excessive pressure. Only 28.1% of respondents felt very slightly about participating in work-related activities during regular working hours. 24.3% of respondents felt slight pressure while complying with departmental and university rules and regulations. Few (20.1%) felt very slight pressure due to having inadequate facilities (office, library, laboratories, classrooms), however, 34.7% of respondents said that making presentations at professional conferences and meetings is not applicable. Similarly, having an insufficient reward for institutional/departmental services is a reason for excessive pressure felt by 9.9% of respondents. Only 32.6% of the respondents said that I have too heavy a workload, one that I cannot possibly finish during the normal workday, which is the reason for very slight pressure. Around one-fifth (17.9%) of respondents said that receiving inadequate salaries to meet financial needs is the reason for excessive stress. Almost a third (31.3%) of respondents said that they feel a slight level of pressure on their working life. Similarly, the same number of respondents are experiencing a slight level of pressure in their daily life.

**Table 1:** Reasons and level of stress felt by respondents (n = 576).

Characteristics	Not applicable	Very slight pressure	Slight pressure	Moderate pressure	High pressure	Excessive pressure
Participating in the work of departmental or university committees	209 (36.3%)	110 (19.1%)	110 (19.1%)	102 (17.7%)	35 (6.1%)	10 (1.7%)
Participating in work-related activities during regular working hours	90 (15.6%)	162 (28.1%)	150 (26.0%)	102 (17.7%)	53 (9.2%)	19 (3.3%)
Complying with departmental and university rules and regulations	87 (15.1%)	129 (22.4%)	140 (24.3%)	115 (20.0%)	68 (11.8%)	37 (6.4%)
Having inadequate facilities (office, library, laboratories, classrooms)	134 (23.3%)	116 (20.1%)	107 (18.6%)	105 (18.2%)	87 (15.1%)	27 (4.7%)
Evaluating the performance of students	60 (10.4%)	139 (24.2%)	112 (19.5%)	123 (21.4%)	83 (14.4%)	58 (10.1%)
Making presentations at professional conferences and meetings	200 (34.7%)	133 (23.1%)	113 (19.6%)	80 (13.9%)	35 (6.1%)	15 (2.6%)
Having students evaluate my teaching performance	130 (22.6%)	191 (33.2%)	115 (20.0%)	79 (13.7%)	42 (7.3%)	19 (3.3%)
Resolving differences with fellow faculty members	125 (21.7%)	162 (28.1%)	118 (20.5%)	95 (16.5%)	55 (9.5%)	21 (3.6%)
Having insufficient time to keep abreast with current developments in my field	95 (16.5%)	122 21.2%	145 (25.2%)	128 (22.2%)	64 (11.1%)	22 (3.8%)
Having insufficient authority to perform my responsibilities	92 (16.0%)	137 (23.8%)	110 (19.1%)	114 (19.8%)	87 (15.1%)	36 (6.3%)
Believing that the progress in my career is not what it should or could be	85 (14.8%)	122 (21.2%)	107 (18.6%)	130 (22.6%)	79 (13.7%)	53 (9.2%)
Preparing a manuscript for publication	180 (31.3%)	120 (20.8%)	102 (17.7%)	91 (15.8%)	58 (10.1%0	25 (4.3%)
Being unclear as to the scope and responsibilities of my job	140 (24.3%)	147 (25.5%)	104 (18.1%)	97 (16.8%)	56 (9.7%)	32 (5.6%)
Having an insufficient reward for institutional/departmental services	125 (21.8%)	106 (18.5%)	88 (15.3%)	110 (19.2%)	88 (15.3%)	57 (9.9%)
Having inadequate time for teaching preparation	139 (24.1%)	134 (23.3%)	112 (19.4%)	111 (19.3%)	56 (9.7%)	24 (4.2%)
Feeling pressure to compete with my colleagues	156 (27.1%)	178 (30.9%)	101 (17.5%)	81 (14.1%)	47 (8.2%)	13 (2.3%)
Having repetition teaching and job assignments	158 (27.4%)	148 (25.7%)	124 (21.5%)	87 (15.1%)	44 (7.6%)	15 (2.6%)
Feeling that I have too heavy a workload, one that I cannot possibly finish during the normal workday	122 (21.2%)	188 (32.6%)	107 (18.6%)	92 (16.0%)	37 (6.4%)	30 (5.2%)
Attending meetings which takes up too much time	124 (21.5%)	135 (23.4%)	129 (22.4%)	90 (15.6%)	52 (9.0%)	46 (8.0%)
Dealing with program changes or reduced enrolment with impact my job	145 (25.2%)	117 (20.3%)	131 (22.7%)	102 (17.7%)	62 (10.8%)	19 (3.3%)
Receiving insufficient recognition for teaching performance.	113 (19.6%)	118 (20.5%)	115 (20.0%)	103 (17.9%)	88 (15.3%)	39 (6.8%)
Making class presentations	112 (19.4%)	177 (30.7%)	125 (21.7%)	90 (15.6%)	47 (8.2%)	25 (4.3%)
Trying to influence my chair’s actions and decisions which affect me	132 (22.9%)	161 (28.0%)	109 (18.9%0	109 (18.9%)	46 (8.0%)	19 (3.3%)
Not having clear criteria for evaluating service activities	111 (19.3%)	113 (19.6%)	110 (19.1%)	96 (16.7%)	97 (16.8%)	49 (8.5%)
Resolving differences with my chair	140 (24.3%)	137 (23.8%)	139 (24.1%)	101 (17.5%)	46 (8.0%)	13 (2.3%)
Lacking congruency in institutional, departmental, and personal goals	122 (21.2%)	134 (23.3%)	127 (22.0%)	77 (13.4%)	73 (12.7%)	43 (7.5%)
Having to teach subject matter for which I am not sufficiently prepared	182 (31.6%)	156 (27.1%)	94 (16.3%)	75 (13.0%)	53 (9.2%)	16 (2.8%)
Receiving insufficient institutional recognition for research performance	158 (27.4%)	112 19.4%	108 (18.8%)	101 (17.5%)	58 (10.1%)	39 (6.8%)
Lacking personal impact on departmental/institutional decision-making	124 (21.5%)	151 (26.2%)	93 (16.1%)	104 (18.1%)	71 (12.3%)	33 (5.7%)
Not knowing how my chair evaluates my performance	115 (20.0%)	130 (22.6%)	120 (20.8%)	101 (17.5%)	71 (12.3%)	39 (6.8%)
Receiving inadequate salary to meet financial needs	105 (18.2%)	115 (20.0%)	82 (14.2%)	78 (13.5%)	93 (16.1%)	103 (17.9%)
Not having clear criteria for evaluation of research and publication activities	160 (27.8%)	132 (22.9%)	72 (12.5%)	79 (13.7%)	71 (12.3%)	62 (10.8%)
Having job demands which interfere with other personal activities (Recreation, family, and other interests)	110 (19.1%)	132 (22.9%)	103 (17.9%)	113 (19.6%)	70 (12.2%)	48 (8.3%)
Being down into conflict between colleagues	177 (30.7%)	147 (25.5%)	94 (16.3%)	89 (15.5%)	48 (8.3%)	21 (3.6%)
Assess the level of stress you experience in your job	0 (0.0%)	122 (21.8%)	175 (31.3%)	156 (27.9%)	75 (13.4%)	31 (5.5%)
Assess the level of stress you experience in your daily life	0 (0.0%)	137 (24.5%)	175 (31.3%)	136 (24.3%)	84 (15.0%0	28 (5.0%)

Table 2 describes the factors of job satisfaction among respondents. Regarding job satisfaction, 19.3% agreed slightly with the statement, *“I feel I am being paid a fair amount for the work I do”*, and a similarly equal percentage of respondents agreed slightly with the statement *“There are very few chances for promotion on my job”*. About one-third (31.9%) of respondents agree moderately with the statement, *“My supervisor is quite competent in doing his/her job*”, and a nearly equal percentage of respondents agree slightly with the statement *“When I do a good job, I receive recognition for it that I should receive”*. Few respondents (15.6%) agree very much with the statement that raises (lift or move to a higher position or level) are too few and far between. Only 6.3% of respondents agree very much with the statement, the goals of this organization are not clear to me. The statement, *“I feel a sense of pride in doing my job”* was agreed very much by 35.2% of respondents, whereas 24.8% of respondents agreed very much with the statement, I like my supervisor. 28.0% of respondents agree slightly with the statement, “*My job is enjoyable”*.

**Table 2:** Factors of job satisfaction among respondents (n = 576).

Characteristics	Disagree very much	Disagree moderately	Disagree slightly	Agree slightly	Agree moderately	Agree very much
I feel I am being paid a fair amount for the work I do.	83 (14.4%)	66 (11.5%)	75 (13.0%)	111 (19.3%)	148 (25.7%)	93 (16.1%)
There is really too little chance for promotion in my job.	74 (12.8%)	68 (11.8%)	61 (10.6%)	110 (19.1%)	106 (18.4%)	157 (27.3%)
My supervisor is quite competent in doing his/her job.	28 (4.9%)	44 (7.6%)	61 (10.6%)	160 (27.8%)	184 (31.9%)	99 (17.2%)
I am not satisfied with the benefits I receive.	51 (8.9%)	86 (14.9%)	90 (15.6%)	152 (26.4%)	135 (23.4%)	62 10.8%
When I do a good job, I receive the recognition for it that I should receive.	69 (12.0%)	71 (12.3%)	91 (15.8%)	167 (29.0%)	108 (18.8%)	70 (12.2%)
Many of our rules and procedures make doing a good job difficult.	42 (7.3%)	86 (14.9%)	85 (14.8%)	171 (29.7%)	122 (21.2%)	70 (12.2%)
I like the people I work with.	13 (2.3%)	15 (2.6%)	30 (5.2%)	117 (20.3%)	206 (35.8%)	195 (33.9%)
I sometimes feel my job is meaningless.	198 (34.4%)	97 (16.8%)	72 (12.5%)	94 (16.3%)	68 (11.8%)	47 (8.2%)
Communications seem good within this organization.	47 (8.2%)	63 (10.9%)	84 (14.6%)	144 (25.0%)	136 (23.6%)	102 (17.7%)
Raises (lift or move to a higher position or level) are too few and far between.	21 (3.6%)	28 (4.9%)	54 (9.4%)	275 (47.7%)	108 (18.8%)	90 (15.6%)
Those who do well on the job stand a fair chance of being promoted.	92 (16.0%)	74 (12.8%)	96 (16.7%)	143 (24.8%)	109 (18.9%)	62 (10.8%)
My supervisor is unfair to me.	172 (29.9%)	127 (22.0%)	96 (16.7%)	104 (18.1%)	50 (8.7%)	27 (4.7%)
The benefits we receive are as good as most other organizations offer.	61 (10.6%)	79 (13.7%)	69 (12.0%)	148 (25.7%)	128 (22.2%)	91 (15.8%)
I do not feel that the work I do is appreciated.	75 (13.0%)	88 (15.3%)	106 (18.4%)	146 (25.3%)	96 (16.7%)	65 (11.3%)
My efforts to do a good job are seldom blocked by red tape (official rules and regulations that seem overly complex and frustrating).	77 (13.4%)	80 (13.9%)	127 (22.0%)	151 (26.2%)	95 (16.5%)	46 (8.0%)
I find I have to work harder at my job because of the incompetence of people I work with.	96 (16.7%)	133 (23.1%)	118 (20.5%)	129 (22.4%)	68 (11.8%)	32 (5.6%)
I like doing the things I do at work.	23 (4.0%)	19 (3.3%)	39 (6.8%)	131 (22.7%)	184 (31.9%)	180 (31.3%)
The goals of this organization are not clear to me.	161 (28.0%)	121 (21.0%)	97 (16.8%)	102 (17.7%)	59 (10.2%)	36 (6.3%)
I feel unappreciated by the organization when I think about what they pay me.	76 (13.2%)	95 (16.5%)	95 (16.5%)	152 (26.4%)	90 (15.6%)	68 (11.8%)
People get ahead(succeed) as fast here as they do in other places.	70 (12.2%)	111 (19.3%)	101 (17.5%)	148 (25.7%)	90 (15.6%)	56 (9.7%)
My supervisor shows too little interest in the feelings of subordinates.	92 (16.0%)	124 (21.5%)	95 (16.5%)	133 (23.1%)	87 (15.1%)	45 (7.8%)
The benefits package we have is equitable.	79 (13.7%)	77 (13.4%)	86 (14.9%)	157 (27.3%)	104 (18.1%)	73 (12.7%)
There are few rewards for those who work here.	77 (13.4%)	73 (12.7%)	65 (11.3%)	159 (27.6%)	106 (18.4%)	96 (16.7%))
I have too much to do at work.	40 (6.9%)	87 (15.1%)	108 (18.8%)	172 (29.9%)	117 (20.3%)	52 (9.0%)
I enjoy my coworkers.	6 (1.0%)	15 (2.6%)	27 (4.7%)	108 (18.8%)	174 (30.2%)	246 (42.7%)
I often feel that I do not know what is going on with the organization.	49 (8.5%)	81 (14.1%)	76 (13.2%)	175 (30.4%)	110 (19.1%)	85 (14.8%)
I feel a sense of pride in doing my job.	9 (1.6%)	20 (3.5%)	46 (8.0%)	127 (22.0%)	171 (29.7%)	203 (35.2%)
I feel satisfied with my chances for salary increases.	77 (13.4%)	60 (10.4%)	80 (13.9%)	155 (26.9%)	123 (21.4%)	81 (14.1%)
There are benefits we do not have that we should have.	35 (6.1%)	54 (9.4%)	67 (11.6%)	173 (30.0%)	123 (21.4%)	124 (21.5%)
I like my supervisor.	16 (2.8%)	37 (6.4%)	46 (8.0%)	139 (24.1%)	195 (33.9%)	143 (24.8%)
I have too much paperwork.	42 (7.3%)	88 (15.3%)	104 (18.1%)	171 (29.7%)	120 (20.8%)	51 (8.9%)
I don’t feel my efforts are rewarded the way they should be.	38 (6.6%)	49 (8.5%)	77 (13.4%)	161 (28.0%)	137 (23.8%)	114 (19.8%)
I am satisfied with my chances for promotion.	114 (19.8%)	63 (10.9%)	69 (12.0%)	212 (36.8%)	76 (13.2%)	42 (7.3%)
There is too much bickering (argue quarrel) and fighting at work.	142 (24.7%)	85 (14.8%)	84 (14.6%)	198 (34.4%))	41 (7.1%)	26 (4.5%)
My job is enjoyable.	18 (3.1%)	23 (4.0%)	51 (8.9%)	161 (28.0%)	189 (32.8%)	134 (23.3%)
Work assignments are not fully explained.	79 (13.7%)	132 (22.9%)	98 (17.0%)	125 (21.7%)	93 (16.1%)	49 (8.5%)

Job satisfaction subscales when compared between government and private college respondents revealed some notable differences (Table 3). Government college employees reported significantly higher levels of satisfaction with pay (mean = 14.97 vs. 13.32, p < 0.001), fringe benefits (mean = 14.42 vs. 13.15, p < 0.001), and nature of work (mean = 18.81 vs. 17.98, p = 0.03). Conversely, private college employees rated supervision significantly higher (mean = 17.22 vs. 16.16, p = 0.005) and co-worker relations (mean = 18.21 vs. 17.03, p = 0.028) more favourably. The groups did not differ significantly in the level of satisfaction with promotion (p = 0.936), contingent rewards (p = 0.34), operating conditions (p = 0.302), and communication (p = 0.73).

**Table 3:** Subscale of job satisfaction among respondents n = 576.

Subscales of job satisfaction	Type of College	Mean ± SD	p-value
Pay	Government	14.97 **± **3.65	<0.001^*^
	Private	13.32 **± **3.94	
Promotion	Government	13.29 **± **4.11	0.936
	Private	13.26 **± **3.86	
Supervision	Government	16.16 **± **4.16	0.005^*^
	Private	17.22 **± **4.16	
Fringe Benefit	Government	14.42 **± **3.54	<0.001^*^
	Private	13.15 **± **3.78	
Contingent Reward	Government	13.49 **± **3.17	0.34
	Private	13.17 **± **3.91	
Operating Conditions	Government	12.87 **± **3.02	0.302
	Private	13.45 **± **2.93	
Co-workers	Government	17.03 **± **3.04	0.028^*^
	Private	18.21 **± **3.37	
Nature of Work	Government	18.81 **± **3.84	0.03^*^
	Private	17.98 **± **3.85	
Communication	Government	14.98 **± **4.51	0.73
	Private	15.11 **± **4.30	

* Significant at <0.05 level.

Table 4 presents that among 576 respondents, 75.3% had a low level of stress, whereas 24.7% had a high level of stress. A majority (62.7%) of the respondents were ambivalent about their job, whereas only less than one-third were satisfied.

**Table 4:** Level of stress among and job satisfaction of the respondents (n = 576).

Characteristics	Level	Frequency	Per cent
Stress level	Low	434	75.3
	High	142	24.7
Job Satisfaction level	Dissatisfied	46	8.0
	Ambivalent	361	62.7
	Satisfied	169	29.3

Table 5 presents the level of stress, and job satisfaction between government and private institutes. While comparing, the level of stress was nearly similar in both government and private institutes. Regarding job satisfaction, 8.9% of respondents in government institutes were dissatisfied with their jobs, whereas 30.7% were satisfied with their jobs in government institutes which was 2% more compared to private institutes. More than half were ambivalent in their job, in private (63.7%) as well as in government institutes (60.3%). Respondents from the government institutes showed a higher level of job satisfaction than the private institutes, but the statistical significance was not established. The levels of stress, and job satisfaction were not significantly different between government and private institutes.

**Table 5:** Level of stress, & job satisfaction among respondents of government and private colleges (n = 576).

Characteristics	Level	Type of college		*χ*^2^ Value	p-value
		Government No (%)	Private No (%)		
Stress Level	Low	140 (78.2)	294 (74.1)	1.48	0.284
	High	39 (21.8)	103 (25.9)		
Job Satisfaction Level	Dissatisfied	16 (8.9)	30 (7.6)	0.692	0.708
	Ambivalent	108 (60.3)	253 (63.7)		
	Satisfied	55 (30.7)	114 (28.7)		

Table 6 shows that the level of stress is not significantly associated with education level, year of experience, income, or job contract status. However, it was significantly associated with the facility and opportunity for higher education by the institution. Stress levels increased when the level of education increased, and the level of stress was not related to the year of job experience or the job contract status.

**Table 6:** Association between the level of stress with selected socio-demographic variables (n = 576).

Characteristics	Categories	Stress level		*χ*^2^ Value	p-value
		Low n (%)	High n (%)		
Education Level	Bachelor	154 (79.4)	40 (20.6)	2.563	0.109
	Master’s and above	280(73.3)	102 (26.7)		
Year of experience	Up to 5 years	200(74.9)	67(25.1)	0.052	0.819
	More than 5 years	234(75.7)	75(24.3)		
The income per month in NRs	< 30000	94(77.7)	27(22.3)	1.345	0.51
	30000 - 59999	267(73.8)	95(26.2)		
	60000+	73(78.5)	20(21.5)		
Opportunity for higher education.	No	212(69.3)	94(30.7)	12.932	<0.001
	Yes	222(82.2)	48(17.8)		
Job contract	Temporary	285(75.2)	94(24.8)	0.013	0.908
	Permanent	149(75.6)	48(24.4)		

Table 7 shows that the job satisfaction level was higher among respondents who had more than 5 years of experience (69.3%), the opportunity for higher education (79.6%), and permanent tenure status (74.6%). The level of job satisfaction was significantly associated with the opportunity for higher education (p < 0.001) and with the job contract status (p = 0.005).

**Table 7:** Association between level of job satisfaction with selected socio-demographic variables (n = 576).

Characteristics	Categories	Satisfaction level		*χ*^2^ Value	p-value
		Low n (%)	High n (%)		
Education Level	Bachelor	55(28.4)	139(71.6)	2.844	0.092
	Master’s and above	135(35.3)	247(64.7)		
Year of experience	Up to 5 years	95(35.6)	172(64.4)	1.516	0.218
	More than 5 years	95(30.7)	214(69.3)		
Income per month. NRs:	< 30000	37(30.6)	84(69.4)	6.853	0.032
	30000 - 59999	132(36.5)	230(63.5)		
	60000+	21(22.6)	72(77.4)		
Opportunity for higher education	No	135(44.1)	171(55.9)	36.593	<0.001
	Yes	55(20.4)	215(79.6)		
Job contract	Temporary	140(36.9)	239(63.1)	7.834	0.005
	Permanent	50(25.4)	147(74.6)		

## Discussion

While there is literature on stress levels and job satisfaction of nurses working in clinical settings, there is little known in this regard about the nurses who work in academic institutions in Nepal. Nepal has a significant private sector involvement in health professions education including nursing education. This study attempts to fill this gap in the literature by assessing the stress and job satisfaction levels among nursing faculty members in academic institutions, especially comparing the government and private colleges in Nepal.

More than half of the respondents belonged to the academic rank of Assistant Professor or Lecturer and the majority were involved in the teaching of Bachelor of Nursing courses. The income range was from NRs 16,000 ($122) to 200,000 per month and the mean income was NRs. 44,454 per month. The minimum salary of $ 122 per month is a very low wage compared to the Government of Nepal’s minimum wage standard. Similar issues of low salaries have been reported in nurses working in clinical settings in the private sector, despite awareness of the issue by the regulatory authorities [23–25,47].

A quarter (24.7%) of the respondents reported a high stress level, which aligns with the findings of a previous study conducted in Biratnagar, Nepal, where 20.4% of the respondents experienced a high level of stress [48] Stress can be the result of organizational, interpersonal, or individual/personal factors and could eventually lead to burnout if not addressed. This study found that the majority (62.7%) of the respondents were ambivalent about their jobs, with less than one-third (29.3%) satisfied. Compared with previous evidence, from Nepal, 36.8% of nursing faculties were satisfied with their current job, [27] while 79% were ambivalent and 21% were dissatisfied [49]. This shows that satisfaction has decreased in this report and further in-depth exploration may be helpful to understand satisfaction. Compared with findings from Oman, it can be seen that job satisfaction levels are not high as they report moderate levels of empowerment and job satisfaction [44].

Organizational facilities may significantly influence stress levels. The study found similar stress levels in both government and private institutes, with government employees being more satisfied and less ambivalent about their jobs compared to private employees. However, the levels of stress and job satisfaction were not significantly different between government and private institutes.

Job satisfaction was significantly associated with opportunities for higher education (p < 0.001) and tenure status (p = 0.005). The study highlighted that opportunities for higher education are crucial determinants of job satisfaction and stress, with government employees having better access to these opportunities compared to those in private institutes.

According to the Nepal Nursing Council, there are 113,376 registered nurses in Nepal, with around 15,000 serving in government health facilities and approximately 17,000 working in the private sector [23,25,50]. Over 20,000 nurses have gone abroad for better opportunities, and many have changed professions due to poor pay, exploitation, and lack of opportunities [23]. Officials from various health organizations have acknowledged the underpayment and exploitation of nurses in the private sector [23,47]. This study found that 13.9% of respondents earned less than NPR 25,000/month, 23.1% worked more than 8 hours/day, 53.1% had no opportunity for educational advancement, and 22% lacked training opportunities to maintain and develop their skills.

The inequitable distribution of health workers in Nepal has led to a critical shortage across most parts of the country. Several nursing positions at state-run healthcare facilities have been unfilled for years. The World Health Organization recommends 2.3 physicians, nurses, and midwives per 1,000 people, which is significantly higher than Nepal’s current ratio of 0.67 [23,47].

The Medical Education Commission (MEC) and the Council for Technical Education and Vocational Training (CTEVT) are responsible for setting and implementing accreditation standards for medical education in Nepal, including nursing education. The production of nursing manpower has declined due to the closure of many colleges that failed to meet MEC criteria. A key criterion among many others to run the Nursing programme at the undergraduate or equivalent level, there must be mandatory 100-bed hospitals with the minimum requirement as mentioned in the MEC regulation [23,51–53].

The successful drafting of a five-year bilateral agreement to deploy 10,000 nurses and other healthcare professionals to the UK offers attractive pay compared to Nepal, which is between NPR 4–4.8 million (~27,000–32,000 GBP) [23]. However, the government views this as a means to welcome remittances rather than focusing on retaining healthcare professionals [54,55]. This culture of health worker mobility has motivated international movement among newcomers [47].

The International Council of Nurses (ICN) emphasizes that quality health care depends on a sufficient supply of qualified, satisfied, and committed nursing personnel. This is supported by the evidence that links good working conditions with quality service provision [50] Countries should determine workers’ general conditions of employment such as minimum leave, occupational health coverage, retirement age, maximum working hours, salary, allowances, and compensation when recruiting the health workforce [47]. ICN condemns unethical hiring procedures that take advantage of or exploit nurses into accepting duties and working conditions that are incompatible with their education, training, and expertise [50].

Many educational institutions lack essential academic environments, such as research publications, teacher evaluations, professional presentations, committee participation, and institutional recognition for research performance. These deficiencies raise questions about the academic standards of institutions. Implementing minimum standards for higher educational institutions is necessary to standardize education quality and maintain a favourable working environment to prevent faculty stress and promote job satisfaction.

Although job satisfaction and stress are abstract factors which is hard to measure by measuring tools and convenient sampling technique was used for data collection it covered a large population, including all provinces of Nepal, compared the academic environment of government and private colleges, and covered the major aspects of jobs, which are needed for faculties retention in academia and promote job satisfaction.

### Strengths and limitations

This study presents several notable strengths. It is the first study of its kind to report on job stress and satisfaction by including all government colleges and a substantial proportion of private colleges in Nepal. Hence the findings are more representative of the nursing workforce. The use of standardized tools for measuring job stress and satisfaction, such as Gmelch’s Faculty Stress Index and Spector’s Job Satisfaction Survey, enhances the reliability and validity of the data collected.

However, the study also has some limitations. The use of convenience sampling for private colleges introduces potential selection bias. Nonetheless, the census approach for government colleges helps mitigate this bias to some extent. As the data is self-reported, there is a possibility of underreporting or overreporting by respondents. To address this, the study ensured confidentiality, anonymity of institutions during data analysis, and autonomy for participants to withdraw at any time, promoting honest responses.

Additionally, while there is a potential for non-response bias due to two out of 41 colleges not participating, this impact is minimal given the high response rate of 96%.

## Conclusions

The study reports that stress levels are high in private colleges and faculties are more satisfied in government colleges. Based on the evidence, the Ministry of Education, Medical Education Commission, universities, and colleges should work together to explore this issue and identify ways to promote a conducive work environment by decreasing stressors and improving job satisfaction among the nursing faculties in Nepal. These include the academic environment, the workload of faculty, facilities, professional development, and research environment, and then should implement standardization strategies to promote and maintain the quality of education in nursing and decrease the stress and burnout of nursing faculties.
